# Heavy metals content in fresh tuna and swordfish caught from Hindian and Pacific Oceans: Health risk assessment of dietary exposure

**DOI:** 10.14202/vetworld.2023.858-868

**Published:** 2023-04-26

**Authors:** Adnorita Fandah Oktariani, Putu Eka Sudaryatma, Yan Ramona, I Made Gelgel Wirasuta, Ida Bagus Gede Darmayasa, Putu Angga Wiradana, Tamaki Okabayashi

**Affiliations:** 1 Doctoral Student of Study Program of Biological Science, Udayana University, Denpasar City, Bali, Indonesia; 2 Fish Quarantine Inspection Agency, Denpasar Bali, Kuta Badung Regency, Bali, Indonesia; 3 Integrated Laboratory of Bioscience and Biotechnology, Udayana University, Jimbaran, Badung Regency, Bali; 4 Study Program of Biology, Faculty of Mathematics and Natural Sciences, Udayana University, Jimbaran, Badung Regency, Bali; 5 Department of Pharmacy, Faculty of Mathematics and Natural Sciences, Udayana University, Jimbaran, Badung Regency, Bali; 6 Study Program of Biology, Faculty of Health, Science, and Technology, Universitas Dhyana Pura, Bali Province, Indonesia; 7 Department of Veterinary Science, Faculty of Agriculture, University of Miyazaki, Miyazaki, Japan; 8 Centre for Animal Diseases Control, University of Miyazaki, Miyazaki, Japan

**Keywords:** health risk assessment, heavy metal pollution, seafood products, Tuna

## Abstract

**Background and Aim:**

Yellowfin tuna and swordfish are seafood commodities commonly caught from deep oceans worldwide. Therefore, this study aimed to assess the levels of three heavy metals, namely, cadmium (Cd), lead (Pb), and mercury (Hg) in yellowfin tuna and swordfish. The results are expected to provide consumers with information on the safety of consuming or exporting these fishes caught in the Hindian and Pacific Oceans.

**Materials and Methods:**

Fresh yellowfin and swordfish were obtained from fishermen’s catches in FAO Fishing Zone 57 (Indian Ocean) and 71 (Pacific Ocean) and then collected at Benoa Harbor, Bali Province. The comparative method was to evaluate the levels of heavy metals in each fish. Furthermore, heavy metal concentrations, including Pb, Cd, and Hg, were determined using atomic absorption spectroscopy analysis. These results were then used to assess the safety of these fishes by calculating the estimated daily intake (EDI) and target hazard quotients-total target hazard quotients (THQs-TTHQs).

**Results:**

The analysis showed that none of the samples exceeded the threshold levels for the three heavy metals, which was specified by the Indonesian National Standard (SNI) and European Commission Regulation (CR) No. 1881/2006. The EDI and provisional tolerable weekly index (PTWI) obtained in this study were still in the safe range. However, the PTWI values for Pb in yellowfin tuna product from the Indian Ocean were higher (0.0038 mg/kg) compared to the recommended standard for the adult population. The THQ-TTHQ values of fish caught from these oceans were also within the acceptable range specified by the two agencies, indicating that they are safe for consumption by people with various age groups and for export purposes.

**Conclusion:**

The average levels of three heavy metals (Cd, Pb, and Hg) in muscle samples of yellowfin tuna and swordfish caught from the Pacific and Hindian Oceans were within the acceptable range as specified by the SNI and CR No. 1881/2006. Furthermore, the EDI and THQs values indicated that fishes caught from the Pacific and Hindian Oceans were safe for consumption. This research is still limited to assessing two capture fisheries commodities. Further research is needed on the assessment of heavy metal levels in other capture fisheries commodities in this capture zone.

## Introduction

In the past two decades, there has been an increase in the consumption of seafood and its derivatives due to high protein and low fat content. Furthermore, these products provide a rich source of nutrition because they contain several essential vitamins and minerals needed by the human body [1, 2]. From a health perspective, omega-3-poly-unsaturated fatty acids have been reported to have beneficial effects on the cardiovascular system, the development of the nervous system in children, particularly during the golden age of growth, as well as prevention of cancer cell progression [3–5]. In several countries, seafood products have been used as alternative food sources to address the food crisis in many countries. They are also considered excellent sources of nutrition, which can be processed into various seafood derivatives due to their high protein content [6]. The previous reports showed that Indonesia has experienced significant growth in the fish and fisheries industries [7] with a focus on the trading of tuna [8].

In Indonesia, yellowfin tuna (*Thunnus albacares*) and swordfish (*Xiphias gladius*) are among the most commonly caught and consumed species of tuna [9]. The global demand for these species has also caused an increase in their catching for export commodities from the Indonesian Ocean [9, 10]. According to projections by the FAO, Indonesia provided up to 20% of the global tuna, skipjack, and tuna output [11]. However, from an ecosystem point of view, these species are among the top predators in the marine ecosystem and tend to accumulate high levels of anthropogenic materials in their body. This can pose a serious risk to human health for those who consume these fishes [12–14]. Heavy metals are common examples of anthropogenic sources that can be found in agricultural wastes, industrial wastes, and oil spills [15, 16]. These types of pollution can become a serious environmental and health problem [17, 18].

Heavy metals have the capacity to accumulate in living tissue, and exposure to them through dietary sources is a global concern [19]. Cadmium (Cd), lead (Pb), and mercury (Hg) have been reported by several studies to accumulate within the body of aquatic organisms. The previous studies have also explored the occurrence of heavy metal contamination in various marine fish species throughout the globe [20–23]. Therefore, it is important to assess the quality of seafood products, such as tuna and tuna-like swordfish, particularly those mostly consumed by humans. This is due to the following factors; (1) different areas of catchment; (2) processing techniques; and (3) techniques of storage that can alter heavy metal concentration in the samples. Indonesia has two important fish catchment areas, namely, FAO Fishing Zones 57 and 71, which are located around the Hindian and Pacific Oceans, respectively [9]. These two areas are also the main routes for shipping, and this has the potential to contribute to anthropogenic source accumulation in the fish obtained.

Therefore, this study aimed to assess the Cd, Pb, and Hg contents of yellowfin and swordfish, caught from the Hindian and Pacific Oceans. The concentration of each type of heavy metal was measured and compared with those previously reported. Based on these results, the ecotoxicological effects of their pollution were determined based on values specified by estimated daily intake (EDI) and target hazard quotients (THQ). The measurements were then used to assess health risk potentials following the consumption of heavy metals-contaminated fish. The results of this study can be used for the early warnings of the marine ecosystem pollution caused these three heavy metals, or as an ideal indicator in the assessment of current marine environmental pollution [24, 25].

## Materials and Methods

### Ethical approval

Ethical clearance was not required for this study because the fish samples were collected from several processing units at Benoa harbor, Bali-Indonesia (for fishes caught from the Indian Ocean) and Bitung, North Sulawesi (for fishes caught from the Pacific Ocean) [9].

### Study period and location

**Figure-1:**
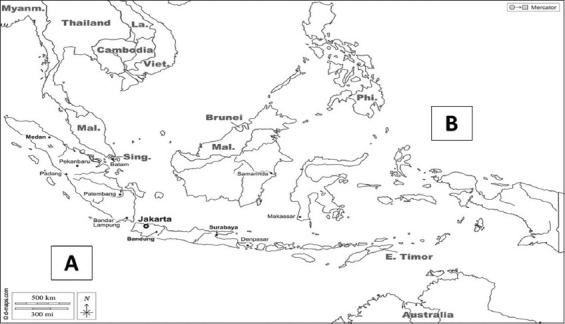
Tuna and swordfish fishing locations. (A) FAO Fishing Zone 57 in the Indian Ocean and (B) FAO Fishing Zone 71 in the Pacific Ocean [Source: www.d-maps.com].

The study was conducted from January 2016 to December 2016. The sampling sites of our study was fish caught from FAO Fishing Zones 57 and 71, which are located around the Indian and Pacific Oceans, respectively, as shown in Figure-1. Furthermore, the fish, particularly yellowfin tuna (*Thunnus albacores*) and Swordfish (*X. gladius*) to be exported to Uni European countries and the USA [9] were subjected to heavy metal analysis at the Fish Quarantine Inspection Agency Denpasar, Bali. A total of 163 fresh fish were obtained from both processing industries between 2012 and 2016. The samples consisted of 40 yellowfin tuna and 36 swordfish from the Pacific Ocean as well as 51 yellowfin tuna and 36 swordfish from the Indian Ocean. The yellowfin tuna and swordfish had an average weight of 90 kg and 100 kg, respectively, and were stored at −32°C before analysis [26].

### Sample preparation

The heavy metal analysis (Pb, Cd, and Hg) was carried out based on the Indonesian National Standard (SNI) method 2354.5.2011. The first stage was the preparation of standard solutions of Pb, Cd, and Hg. These included a primary standard solution of 1000 mg/L; first, second, and third secondary standard solutions of 10 mg/L, 1 mg/L, and 100 μg/L; standard solution in the reading range of the atomic absorption spectroscopy (AAS) machine; and a blank solution. Furthermore, to prepare the fish for analysis, a total of 1 g of fresh samples were ground and homogenized in a grinding machine. The prepared samples were then stored in sterile polystyrene containers and kept or stored in a freezer until they were required in the analysis.

### Analysis of total Cd, Pb, and Hg

The previously prepared samples were wet-destructed in a microwave oven. A wet sample weighing 0.5 g was transferred into a sample tube, while a Certified Reference Material-DORM of 0.25 g served as the control. These tubes were then sequentially added with 8 mL of 65% nitric acid, and 2 mL of 2% H_2_O_2_. Furthermore, sample destruction was carried out by adjusting the program as specified in the digestion manual of the microwave. The product was then transferred into a destruction flask and its volume was adjusted up to 50 mL by adding deionized water. The Pb, Cd, and Hg levels were measured using the AAS machine at the wavelength of 283 nm, 288 nm, and 253 nm, respectively. The results obtained were plotted on the standard curve, which was previously established. In the case where the reading was higher than 0.8, the samples were further diluted when the reading exceeded 0.8 to ensure the value obtained was within the range of the standard curve. The formula below was used to calculate heavy metal levels:


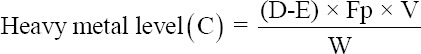


where D is the AAS value of the heavy metal concentration (μg/L), E is AAS value of blank (μg/L), V is final volume of prepared samples (mL), Fp is dilution factor, and W is sample wet weight (g).

### Public health risk assessment of heavy metals in fish sample

#### Estimated daily intake (EDI)

Estimated daily intake of Cd, Pb, and Hg was analyzed based on the average content of heavy metals in each fish product consumed daily by people. The EDI value was then calculated using the following formula [27–29]:


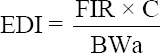


where FIR = rate of daily fish consumption (g/person/days), C = average heavy metal content of sample (mg/kg), and BWa = average body weight. Body weights of 10 kg, 15 kg, 20 kg, 44 kg, and 78 kg were used in the calculation of EDI for people aged 1, 3, 6, and 12 years, as well as adults, respectively. Furthermore, total consumption of yellowfin tuna and swordfish meat for people aged 1, 3, 6, and 12 years, as well as adults was 0.681 g/person, 1.498 g/person, 3.196 g/person, and 3.140 g/person, respectively. The classification was carried out based on the level of consumption of tuna or processed tuna or seafood products, as specified by Núñez *et al*. [26].

#### Provisional tolerable weekly index (PTWI)

The PTWI value is used to emphasize the weekly exposure to heavy metals in food consumed for contaminants that accumulate in the body. The PTWI value of dietary intake of tuna and swordfish meat containing Pb, Cd, and Hg is calculated using the following equation:


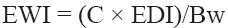


Where C = concentration of heavy metals in seafood, EDI = daily consumption rate (g/day) of seafood, and bw = body weight (age 1 year = 10 kg, age 3 years = 15 kg, age 6 years = 20 kg, age 12 years = 44 kg, and age of the adult population = 78 kg). The estimated weekly intake was compared with the temporarily tolerable weekly intake value of each heavy metal (Hg = 0.004 mg/kg, bb/week; Cd = 0.007 mg/kg, bw/week; and Pb = 0.0025 mg/kg, bw/weeks) [30].

#### Determination of target hazard quotients-total target hazard quotients (THQs-TTHQs)

The THQ is a ratio of the pollutant’s measured value to the dose level (references of oral dose). This ratio was used as an indicator of health risk assessment that can occur as well as in the determination of the carcinogenic level of food samples. Furthermore, if the value of THQ was <1, the food samples have no side effects on people after exposure. This risk calculation was based on the assumption that heavy metals at such concentration can totally be absorbed and their concentration was not affected by cooking processes [31]. The formula below was used in the calculation of THQ [32–35]:


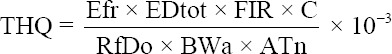


Where Efr = frequency of exposure (365 days/year), EDtot = exposure period (adult: 70 years old, children of 1, 3, 6, and 12 years old), FIR = intake food level (g/day), C = average level of Cd, Pb, or Hg in fish tissues (ppm), RfDo = references of oral dose (Cd = 0.001 mg/kg; Pb = 0.0035 mg/kg; and Hg = 0.0001 mg/kg) [36, 37], Bwa = average body weight based on age groups (70 kg for adult, 10 kg, 15 kg, 20 kg, and 44 kg for children of 1, 3, 6, and 12 years old, respectively), and ATn = average exposure period on non-carcinogenic (71.5 years), based on average Indonesian people’s life expectancy.

The TTHQ obtained in this study was used to assess the bad side effects of exposure to two or more types of heavy metals. The TTHQ value was a sum of the THQ of each heavy metal, using the following formula [38, 39]:


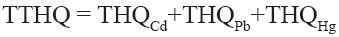


The higher the TTHQ value, the bigger the toxic effect that can occur [40].

### Statistical analysis

The level of heavy metal in the fish samples, caught from the two sites of catchment, was statistically analyzed using t-test analysis (at p < 0.05) with statistical package for the social sciences 23.0 (IBM Corp., USA) software. Furthermore, graphs were generated using GraphPad software Version 8.0 (GraphPad, USA), and the values of EDI, estimated dietary intake (EWI), THQ, and TTHQ were tabulated and analyzed using software MS Excel 2019 (Microsoft, USA) [22].

## Results and Discussion

### Heavy metals contents in yellowfin tuna and swordfish

**Figure-2:**
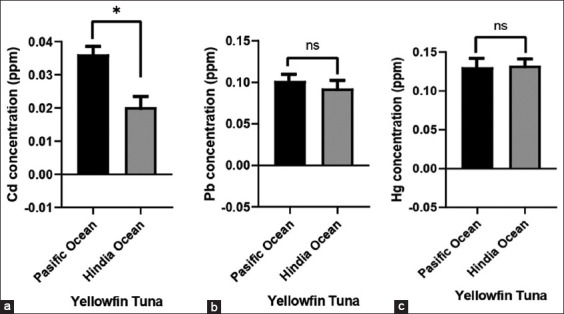
Average levels of (a) cadmium (Cd), (b) lead (Pb), and (c) mercury (Hg) of muscles of yellowfin tuna (*Thunnus albacares*), caught from Pacific and Hindian Oceans. The heavy metal concentrations of samples were compared with that specified in the national standard quality control of Indonesia (SNI) No. 2354.5 year 2011 (for Pb and Cd); SNI No. 2356.6 year 2015 for Hg; and the European Commission Regulation No. 1881/2006 regarding setting maximum levels for certain contaminants in foodstuffs.

The levels of Cd, Pb, and Hg in the muscles of fresh yellowfin tuna used in this study are presented in Figure-2. The results showed that the Cd level in samples from the Pacific Oceans (0.036 mg/kg) was almost two folds higher (p < 0.05) compared to those from Indian Ocean (0.020 mg/kg), as shown in Figure-2a. Furthermore, the Pb and Hg in yellowfin tuna samples from the Pacific Ocean were also higher compared to those from the Hindian Ocean, but they are not statistically significant (p > 0.05), as shown in Figures-2b and c. Based on the SNI regulation for 2011 and 2015 and the CR No. 1881/2006 regarding setting maximum levels for certain contaminants in foodstuffs, the measured heavy metal contents in yellowfin tuna in this study were still within the acceptable ranges.

**Figure-3:**
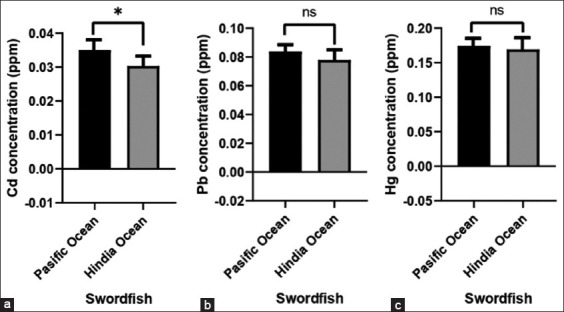
Average levels of (a) cadmium (Cd), (b) lead (Pb), and (c) mercury (Hg) of muscles of swordfish (*Xiphias gladius*), caught from Pacific and Hindian oceans. The heavy metal concentrations of samples were compared with that specified in the national standard quality control of Indonesia (SNI) No. 2354.5 year 2011 (for Pb and Cd); SNI No. 2356.6 year 2015 for Hg; and the European Commission Regulation No. 1881/2006 regarding setting maximum levels for certain contaminants in foodstuffs.

Figure-3(a-c) shows the levels of Cd, Pb, and Hg in the swordfish (*X. gladius*) caught from the Indian and Pacific Oceans. The levels of these metals in samples caught from the Pacific Ocean (p < 0.05) were higher compared to those from the Indian Ocean. The swordfish (*X. gladius*) obtained from these areas of catchment contained approximately the same amount of Hg, but was not statistically significant (p > 0.05), as shown in Figure-3c. Furthermore, based on the quality standards specified by the SNI in 2011 and 2015, as well as the CR No. 1881/2006 regarding setting maximum levels for certain contaminants in foodstuffs, the levels of these three heavy metals in both yellowfin tuna and the swordfish were still within the acceptable levels.

The results provided an overview of the food safety monitoring of seafood products exported from Indonesia to other countries, particularly European countries. This study only measured the levels of heavy metals (Cd, Pb, and Hg) in two types of fish mostly caught from the Pacific and Indian Oceans without considering their size; hence, further studies are needed to examine the effect of fish sizes on the heavy metal contents. Stamatis *et al*. [41] reported that fishes with higher average size in terms of length and weight tended to contain higher heavy metals compared to those with lower sizes. Furthermore, it has been reported in the past three decades that there was a positive correlation between Hg content and the size of bluefin tuna (*Thunnus thynnus*) caught from the Mediterranean Ocean, as well as yellowfin tuna (*T. albacares*) and cakalang (*Katsuwonus pelamis*) from west Hindian Ocean [42, 43].

In this study, a higher level of Cd was obtained in yellowfin tuna and swordfish caught from the Pacific Oceans compared to those from the Hindian partly, due to different conditions of the geographic areas. Other factors, such as fish species, age of the fish, sex, as well as types of diets can contribute to the accumulation of the metal in tuna fish [44, 45]. Cadmium is a heavy metal with a relatively low distribution at sea level, because it was mostly absorbed by phytoplankton [46, 47]. Furthermore, its existence in nature was affected by human activities in various industrial sectors, such as the combustion of fossil fuels and non-ferrous metal extraction. The presence of several Cd pollution sources in marine environments can also be attributed to industrial applications, such as corrosive reagents, as well as their use as stabilizers in Polyvinyl chloride (PVC) products, color pigments, and nickel-Cd batteries, which are currently being developed in large quantities for electrical-based vehicles [48]. The Cd level in yellowfin tuna and swordfish in this study was generally lower compared to the threshold values specified by the SNI (0.10 mg/kg) and CR as 0.10 mg/kg and 0.05 mg/kg, respectively. The results of this study are inconsistent with the previous studies where the levels of Cd in *T. albacores* and *Coryphaena hippurus* caught from the Ecuadorian Ocean exceeded the threshold values specified by the above agencies, namely, 0.011–17 mg/kg and 0.011–11 mg/kg [49], respectively. Table-1 shows the comparison of Cd, Pb, and Hg levels in various seafood products caught from different regions of the catchment [26, 49–54].

**Table-1:** Comparison of Cd, Pb, and Hg concentrations (mg/kg of wet weight) in fresh seafoods including tuna caught from various regions of catchment.

S. No.	Species	Location	Heavy metal levels (mg/kg of wet weight)			References

			Cd	Pb	Hg	
1.	*Thunnus albacares*	Ecuadorian coast (Eastern Pacific)	(<0.011–17)	(<0.04–0.32)	(<0.005–6.0)	[49]
2.	*Coryphaena hippurus*	Ecuadorian coast (Eastern Pacific)	<0.011–11	<0.04–1.86	0.04–5.3	[49]
3.	*Thunnus alalunga*	Galicia Market, NW Spain	ND–0.036	ND–0.025	-	[26]
4.	*Sardina pilchardus*	Algerian coast	<0.01–2.11	0.51–7.53	0.15–0.89	[50]
5.	*Xiphias gladius*	Algerian coast	0.018–7.52	0.02–11.40	0.27–0.88	[50]
6.	*Thunnus obesus*	Atlantic Indian Oceans	0.001–0.136 0.004–0.148	-	-	[51]
7.	*Thunnus alalunga*	Western and Central Pacific Ocean	-	-	0.239–1.180	[52]
8.	*Thunnus obesus*	Western and Central Pacific Ocean	-	-	0.158–3.324	[52]
9.	*Stolephorus indicus*	UAE, coast, Arabian Gulf	0.1–8.0	-	0.04–0.18	[53]
10.	*Seriola dumerili*	Mediterranean Sea	0.122	0.211	-	[54]
11.	*Thunnus albacares*	Pacific Oceans Hindian Oceans	0.006–0.387 0.000–0.074	0.024–0.189 0.000–0.159	0.100–0.352 0.005–0.235	This study
12.	*Xiphias gladius*	Pacific Oceans Hindian Oceans	0.006–0.239 0.000–0.185	0.004–0.168 0.000–0.120	0.020–0.569 0.025–0.442	This study
Quality standards			0.10	0.20	1.0	SNI and CR
			0.05	0.30	1.0

Cd=Cadmium, Pb=Lead, Hg=Mercury, SNI=Indonesian National Standard, CR=European Commission Regulation

The average Pb and Hg concentrations in this study were not significantly different in both fish species caught from the Pacific and Indian Oceans, as shown in Table-1. However, these values were still within the acceptable range specified by the SNI and CR. The results of this study are in line with that of Abolghait and Garbaj [55], where the levels of Hg, Cd, and Pb in canned tuna, marketed in Tripoli, were 0.163 mg/kg, 0.027 mg/kg, and 0.075 mg/kg, respectively. They also reported the presence of a high Hg concentration of 1.185 mg/kg in products of fresh small tunny (*Euthynnus alletteratus*) captured from the Mediterranean Ocean, which exceeded the threshold value. Abolghait and Garbaj [55], revealed a significantly higher level of Hg in big-eye tuna compared to albacore tuna as reported by Houssard *et al*. [56]. The concentration of Hg in *Sardina pilchardus* and *X. gladius* obtained from the Algerian coast was also reported by Genchi *et al*. [48] to be within the acceptable range. Meanwhile, the Pb concentration in these seafood products was reported to be higher than the standard, namely, 0.51–7.53 mg/kg and 0.02–11.40 mg/kg for *S. pilchardus* and *X. gladius*, respectively. A recent study by Ruelas-Inzunza *et al*. [57] showed that Hg and arsenic (As) levels in skipjack tuna from the east Pacific were still within the standard levels, as specified by the CR.

Although the Cd, Pb, and Hg that accumulated in the muscles of yellowfin tuna and swordfish in this study were within the acceptable range for consumption; further studies are still needed. This was because the concentrations of these heavy metals depended on various factors, including the process of ingestion/assimilation, environmental conditions, fish size, fish species, and the accumulation capability of organs, such as the liver, gills, and muscle [58, 59]. The liver of fish with the capability to accumulate higher pollutants, including heavy metals compared to other organs, such as gills and muscle, has been considered a bioindicator of pollution after a short period of exposure [60, 61].

### Toxicological repercussions

#### Estimated daily intake

The EDI value for each heavy metal (Cd, Pb, and Hg) in the different age groups was within the tolerable daily intake range, as shown in Tables-2 and 3 [26, 62]. Therefore, yellowfin tuna and swordfish caught from the Pacific and Indian Oceans were safe for consumption. This indicated that the long-term consumption of such products does not have a chronic effect if the levels of heavy metal contamination remain stable over time.

**Table-2:** Estimated daily intakes of Cd, Pb, dan Hg (mg/kg of wet weight) by children and adulthood following consumption of yellowfin tuna and sword fish caught from Pacific oceans.

Age	Fish ingestion rate (FIR) fresh (kg/person)^1^	Cd	Pb	Hg
Yellowfin Tuna				
Age 1	0.681	0.002	0.007	0.009
Age 3	1.498	0.004	0.010	0.013
Age 6	1.498	0.003	0.008	0.010
Age 12	3.196	0.003	0.007	0.009
Adults	3.14	0.002	0.005	0.006
Swordfish				
Age 1	0.681	0.002	0.006	0.012
Age 3	1.498	0.004	0.008	0.017
Age 6	1.498	0.003	0.006	0.013
Age 12	3.196	0.003	0.006	0.013
Adults	3.14	0.002	0.004	0.008
TI^2^		0.830	3.570	0.300

1 Daily FIR (kg/person) based on [26]. ^2^Tolerable intake suggested by [62]. Cd=Cadmium, Pb=Lead, Hg=Mercury, FIR=Fish ingestion rate

**Table-3:** Estimated daily intakes of Cd, Pb, and Hg (mg/kg of wet weight) by children and adulthood following consumption of yellowfin tuna and sword fish caught from caught from Hindian ocean.

Age	FIR fresh (kg/person)^1^	Cd	Pb	Hg
Yellowfin Tuna				
Age 1	0.681	0.001	0.007	0.009
Age 3	1.498	0.002	0.010	0.013
Age 6	1.498	0.002	0.007	0.010
Age 12	3.196	0.002	0.007	0.010
Adults	3.14	0.001	0.004	0.006
Swordfish				
Age 1	0.681	0.002	0.006	0.012
Age 3	1.498	0.003	0.009	0.017
Age 6	1.498	0.003	0.007	0.013
Age 12	3.196	0.002	0.006	0.012
Adults	3.14	0.002	0.004	0.008
TI^2^		0.830	3.570	0.300

1 Daily FIR (kg/person) based on [26]. ^2^Tolerable intake suggested by [62]. Cd=Cadmium, Pb=Lead, Hg=Mercury, FIR=Fish ingestion rate

Cadmium

In this study, no threat indication due to Cd contamination of fish caught from the Pacific and Indian Oceans was observed. This was because the level of the metal was still lower than the acceptable range specified by Joint FAO/WHO Expert Committee on Food Additives (JECFA) [62]. Furthermore, the daily tolerable Cd intake per kg body weight, specified by this agent, was 0.83 g. This indicated that yellowfin tuna and swordfish caught from Pacific and Hindian Oceans were safe for consumption, as they contained a low level of Cd. Ormaza-González *et al*. [63] reported that the highest accumulation of heavy metals, including Cd, occurred in the liver of tuna fish. However, this part was rarely consumed, indicating that its adverse effect was negligible.

Lead

The EDI value of the samples was lower than the range specified by JECFA [62], namely, <3.57 g/kg body weight/day, indicating that it was negligible. The existence of heavy metals in the environment was due to the disposal of wastes of battery and weapon manufacturers, as well as oil spills [64]. The analysis of Pb in fish samples caught from Indian and Pacific Oceans showed that the concentration of the heavy metal was significantly lower compared to the range specified by SNI and JECFA. Based on the value obtained, the fish products caught from these two oceans were still safe for consumption by people. However, several disorders, such as anemia, malfunction of the brain and kidney, or death, can occur if the level increases over time with long-term exposure [65]. A previous study reported that Pb has the potential to cross the barrier of the placenta and cause damage or inhibit the development of the embryo’s nervous system [66].

Mercury

Methylmercury (MeHg) is a toxic material with negative effects on human health. Mercury also has a toxic effect on the development of the human nervous system, and it is similar to Pb. Furthermore, it commonly accumulates in the muscle of fish, and consumption of Hg-containing fish muscle can Pb to its bioaccumulation [67]. Tables-2 and 3 show the EDI values of yellowfin tuna and swordfish caught from the Pacific and Indian Oceans, which were still within the acceptable range specified by SNI and JECFA. According to the JECFA [62], the maximum Hg daily food exposure was 0.300 g/kg. Mercury can naturally be obtained from a volcano eruption, where it precipitates on the seabed after the biogeochemical cycle. This hypothesis is in line with the finding of Hg content in tuna fish caught from the Azorean Ocean, which was hypothesized to be closely related to the volcanic region around this area [68]. Other anthropogenic sources of Hg included coal used in electrical power generation [69], mining activities [23], and combustion of fossil fuel [70].

#### Provisional tolerable weekly intake (PTWI)

The measurement results of the EWI values for yellowfin tuna and swordfish collected from the Pacific Ocean and Indian Ocean in this study were still below the minimum limit values for Pb, Cd, and Hg based on PTWI. On average, the EWI value for Cd did not differ between the two fish samples caught from the Pacific Ocean, namely, 0.000005 mg/kg (<0.007 mg/kg, PTWI for Cd). However, the average value for Pb metal found in yellowfin tuna from the site was higher (0.000040 mg/kg) compared to swordfish (0.000028 mg/kg). The value obtained was also still safe when compared to the recommended PTWI for Pb, namely, <0.0025 mg/kg. The EWI for Hg metal in swordfish from the Pacific Ocean was higher (0.000108 mg/kg) compared to yellowfin tuna (0.000066 mg/kg) and was still in a safe range based on PTWI, namely, <0.004 mg/kg, as shown in Table-4 [26].

**Table-4:** Estimated dietary intake values of heavy metals (Pb, Cd, and Hg) in yellowfin tuna and swordfish caught from the Pacific Ocean.

Age	Cd	Pb	Hg
Yellowfin Tuna			
Age 1	0.000009	0.00007	0.000116
Age 3	0.000009	0.00007	0.000113
Age 6	0.000005	0.00004	0.000064
Age 12	0.000002	0.00002	0.000028
Adults	0.000001	0.00001	0.000010
Swordfish			
Age 1	0.000008	0.000048	0.000207
Age 3	0.000008	0.000047	0.000152
Age 6	0.000005	0.000032	0.000114
Age 12	0.000002	0.000012	0.000050
Adults	0.000001	0.000004	0.000018

PTWI standard values for each metal, respectively, Cd=0.007 mg/kg; Pb=0.0025 mg/kg; and Cd=0.007. Standard values based on [26]. Cd=Cadmium, Pb=Lead, Hg=Mercury

The EWI value of Pb metal in yellowfin tuna from the Indian Ocean was 0.0038 mg/kg and this exceeded the PTWI recommendation of 0.0025 mg/kg, especially for the adult population, as shown in Table-5 [26]. Meanwhile, the value for other age groups was still within the safe range for consumption when compared to the PTWI value in this study. The EWI of swordfish caught from the Indian Ocean has a safe consumption value compared to the PTWI value in this study.

**Table-5:** Estimated dietary intake values of heavy metals (Pb, Cd, and Hg) in yellowfin tuna and swordfish caught from the Hindian Ocean.

Age	Cd	Pb	Hg
Yellowfin Tuna			
Age 1	0.000063	0.0000030	0.000120
Age 3	0.000062	0.0000030	0.000117
Age 6	0.000035	0.0000017	0.000066
Age 12	0.000015	0.0000007	0.000029
Adults	0.003882	0.0000003	0.000010
Swordfish			
Age 1	0.000052	0.000008	0.000195
Age 3	0.000051	0.000008	0.000191
Age 6	0.000028	0.000004	0.000107
Age 12	0.000013	0.000002	0.000037
Adults	0.000004	0.000001	0.000016

PTWI standard values for each metal, respectively, Cd=0.007 mg/kg; Pb=0.0025 mg/kg; and Cd=0.007. Standard values based on the study by Núñez *et al*. [26]. Cd=Cadmium, Pb=Lead, Hg=Mercury

Similar studies showed that swordfish caught from the waters of FAO zone area 37, division 37.2.2 collected at an Italian fish trading company had a high PTWI. High values for MeHg and Hg were obtained in samples measuring 138–233 cm and 167–233 cm, respectively [30]. The results revealed that fish size influenced the bioaccumulation capacities for heavy metals, and this had an impact on the PTWI value also referred to as the weekly consumption limit. The values obtained in this study were lower compared to the previous studies, which were higher at 0.47 mg/kg body weight/w for As, and 0.05 mg/kg body weight/w for Cd, Hg, and Pb [71]. It is important to note that the EWI of Pb metal found in this study, especially for adult populations of yellowfin tuna from the Indian Ocean, exceeded the PTWI’s recommendations. This indicated that comprehensive monitoring efforts are still needed for this type of metal in other capture fisheries commodities.

#### Target hazard quotients

The THQ value for children and adults in this study was lower than 1, as shown in Table-6. Based on the results, none of the fish samples caught from Pacific and Hindian Oceans analyzed had THQ values for Hg, Pb, and Cd higher than 1, indicating that they were safe for consumption. Meanwhile, samples obtained from North Aegean Sampling Station Area (NASSA) and Southeastern Aegean Sampling Station Area (SASSA) have been reported to have THQ for Hg of >1 in albacore tuna (*Thunnus* *alalunga*), which were within the range of 1.338–5.040 and 0.758–4.046, respectively [41]. A study by Han *et al*. [72] reported that the value obtained in seafood products caught in the Taiwan Ocean for Cd and Hg was lower than 1 (<1), but higher than 1 for inorganic As, Copper, and Zinc in adults. A recent study by Djedjibegovic *et al*. [73] showed that bluefin tuna and mackerel from Bosnian and Herzegovinian Oceans had THQ for Hg of close to 1. This became a great concern after the consumption of such products in moderate quantities particularly by adults and pregnant women. Although the THQ index analysis was not fully appropriate to provide harmful health impacts on consumers, this methodology was important to reveal initial information on the potential risks that can occur after the consumption of heavy metal-contaminated seafood products [74, 75].

**Table-6:** Value of target hazard quotients on yellowfin tuna and swordfish caught from Pacific and Hindian oceansage of consumers.

Heavy metals	Age	Pacific Ocean		Hindia Ocean	
	
		Yellowfin Tuna	Swordfish	Yellowfin Tuna	Swordfish
Cd	Age 1	6.4E + 03	6.42E + 03	3.74E + 03	6.04E + 03
	Age 3	6.4E + 04	6.36E + 04	3.71E + 04	5.98E + 04
	Age 6	1.7E + 05	1.70E + 05	9.88E + 04	1.59E + 05
	Age 12	1.6E + 06	1.59E + 06	9.28E + 05	1.50E + 06
	Adults	1.5E + 07	1.45E + 07	8.46E + 06	1.36E + 07
Pb	Age 1	5.2E + 03	5.17E + 03	4.90E + 03	4.44E + 03
	Age 3	5.1E + 04	5.12E + 04	4.85E + 04	4.39E + 04
	Age 6	1.4E + 05	1.37E + 05	1.29E + 05	1.17E + 05
	Age 12	1.3E + 06	1.28E + 06	1.21E + 06	1.10E + 06
	Adults	2.0E + 06	1.17E + 07	1.11E + 07	1.00E + 07
Hg	Age 1	2.3E + 05	2.32E + 05	2.36E + 05	3.01E + 05
	Age 3	2.3E + 06	2.30E + 06	2.33E + 06	2.98E + 06
	Age 6	6.1E + 06	6.12E + 06	6.22E + 06	7.94E + 06
	Age 12	5.7E + 07	5.75E + 07	5.84E + 07	7.45E + 07
	Adults	5.2E + 08	5.24E + 08	5.32E + 08	6.80E + 08

Cd=Cadmium, Pb=Lead, Hg=Mercury

#### Total target hazard quotients

In this study, the value of TTHQs in the yellowfin tuna and swordfish samples was still within the acceptable range or lower than 1, as shown in Table-7. The TTHQs value covered the average of all heavy metals analyzed. Based on these results, fishes caught from Hindian and Pacific Oceans were safe for consumption by all age groups. Furthermore, the values obtained in this study were lower than those reported by Stamatis *et al*. [41], who analyzed albacore tuna from NASSA and SASSA Aegea (Greek Oceans), namely, 1.353 and 5.21, respectively. This explainsthat consumers of such products have high health risks due to the consumption of these seafoods. Total target hazard quotients values of > 1 were also reported by Li *et al*. [76] after analyzing wild fishes caught from Liuzhou river, China, namely, 1.627 among children.

**Table-7:** Value of total target hazard quotients in yellowfin tuna and swordfish caught from Pacific and Hindian oceans, based on age of their consumers.

Locations	Sample	Age	TTHQs
Pacific Ocean	Yellowfin Tuna	Age 1	2.4E + 05
		Age 3	2.4E + 06
		Age 6	6.4E + 06
		Age 12	6.0E + 07
		Adults	5.4E + 08
	Swordfish	Age 1	2.44E + 05
		Age 3	2.41E + 06
		Age 6	6.43E + 06
		Age 12	6.03E + 07
		Adults	5.50E + 08
Hindia Ocean	Yellowfin Tuna	Age 1	2.44E + 05
		Age 3	2.42E + 06
		Age 6	6.44E + 06
		Age 12	6.05E + 07
		Adults	5.52E + 08
	Swordfish	Age 1	3.11E + 05
		Age 3	3.08E + 06
		Age 6	8.22E + 06
		Age 12	7.71E + 07
		Adults	7.03E + 08

TTHQs=Total target hazard quotients

## Conclusion

This study showed the average levels of three heavy metals, namely, Cd, Pb, and Hg in muscle samples of yellowfin tuna and swordfish caught from the Pacific and Indian Oceans. Furthermore, the values obtained were still in the range of acceptable levels as specified by SNI and CR No. 1881/2006. The EDI and THQs for the three metals indicated that fishes caught from the Pacific and Indian Oceans were safe for consumption purposes. This showed that seafood products harvested from these areas were safe for exporters and there was no health risk for consumers. These findings are consistent with the TTHQs value obtained for the two regions. However, further risk assessment is required, particularly on the seafood products consumed by children and pregnant women.

## Authors’ Contributions

AFO, YR, PAW, and PES: Conceptualization. AFO, PAW, and TO: Methodology. PAW and AFO: Software and formal analysis. YR, IMGW, IBGD, and TO: Validation. AFO, PES, and PAW: Investigation. AFO, PAW, PES, and YR: Data curation. PAW, PES, and AFO: Writing-original draft preparation. PAW, YR, and TO: Writing review and editing. PAW, AFO, and PES: Visualization. YR, IMGW, and IBGD: Supervision. All authors have read, reviewed, and approved the final manuscript.
